# Determination of Sb(III) and Sb(V) by HPLC—Online isotopic dilution—ICP MS

**DOI:** 10.1016/j.mex.2015.12.001

**Published:** 2015-12-12

**Authors:** Maria Chiara Fontanella, Gian Maria Beone

**Affiliations:** Università Cattolica del Sacro Cuore, Istituto di Chimica Agraria e Ambientale, Via E. Parmense 84, 29100 Piacenza, Italy

**Keywords:** Trivalent antimony, Pentavalent antimony, Diffusive gradient in thin film, High Performance Liquid Chromatography–Inductively Coupled Plasma Mass Spectrometer, Water, Isotopic dilution

## Abstract

This work provides a method with application of valid techniques to extract and determinate inorganic species of antimony (Sb) for water. The procedure involves

• the simultaneous accumulation of Sb(III) and Sb(V) on passive samplers like Diffusive Gradient in Thin Films (DGT) with iron (Fe) oxide gel, eliminating the risk of speciation changes due to transport and storage;
• application of less concentrated acid (50 mM Na_2_EDTA) for elution and preservation of Sb species from DGT resin;
• subsequent analytical determination of inorganic species with High Performance Liquid Chromatography–Isotopic Dilution–Inductively Coupled Plasma Mass Spectrometer (HPLC-ID-ICP MS) based on determination of the isotope ratio (^123^Sb/^121^Sb) of isotopes in the samples after spiking with 123Sb enriched standard solution, reducing the effect of signal drift and matrix effect on the final value.

## Graphical abstract

**Figure:**
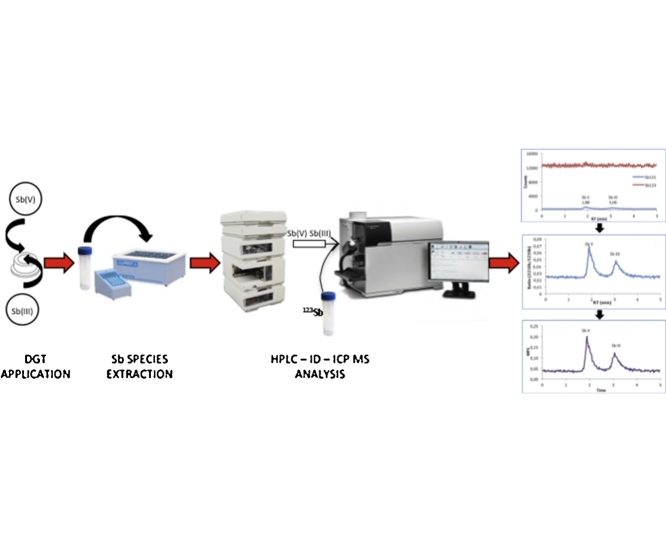

## Method details

Most of the analytical methods for antimony assessment are based on the determination of total antimony concentrations. However, it is widely accepted that the impact of a toxic element on the environment is linked to the presence of its chemical forms [1].

Coupled techniques, based on the combination of a separation method with a suitable element adsorption system, have become reliable in speciation analysis to discriminate specific forms of an element.

We report the first investigation of use of Fe-oxide gels in Diffusive Gradient in Thin Films (DGT) for incorporation of inorganic chemical forms of antimony, especially Sb(III), coupled with High Performance Liquid Chromatography–Isotopic Dilution–Inductively Coupled Plasma mass spectrometer (HPLC-ID-ICP MS).

## Development of method

**Fig. 1:**
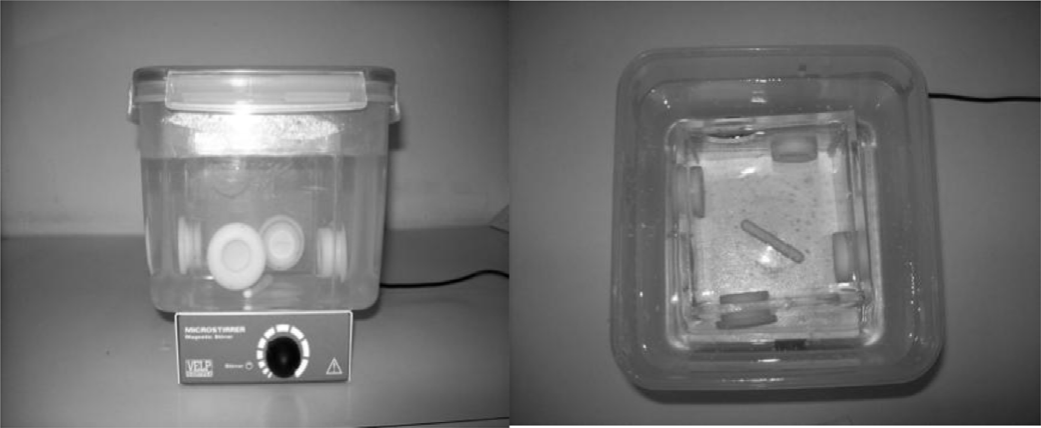
Aqueous solution with Sb(III) and Sb(V) well stirred at constant temperature.

To develop the method, test of kinetics of binding and elution efficiency of less concentrated acid (50 mM Na_2_EDTA) were applied to ensure an appropriate quantitative recovery of the element from the resin gel of DGT (see SUPPLEMENTARY INFORMATION, Table 1S and Fig. 1S).

**Fig. 2:**
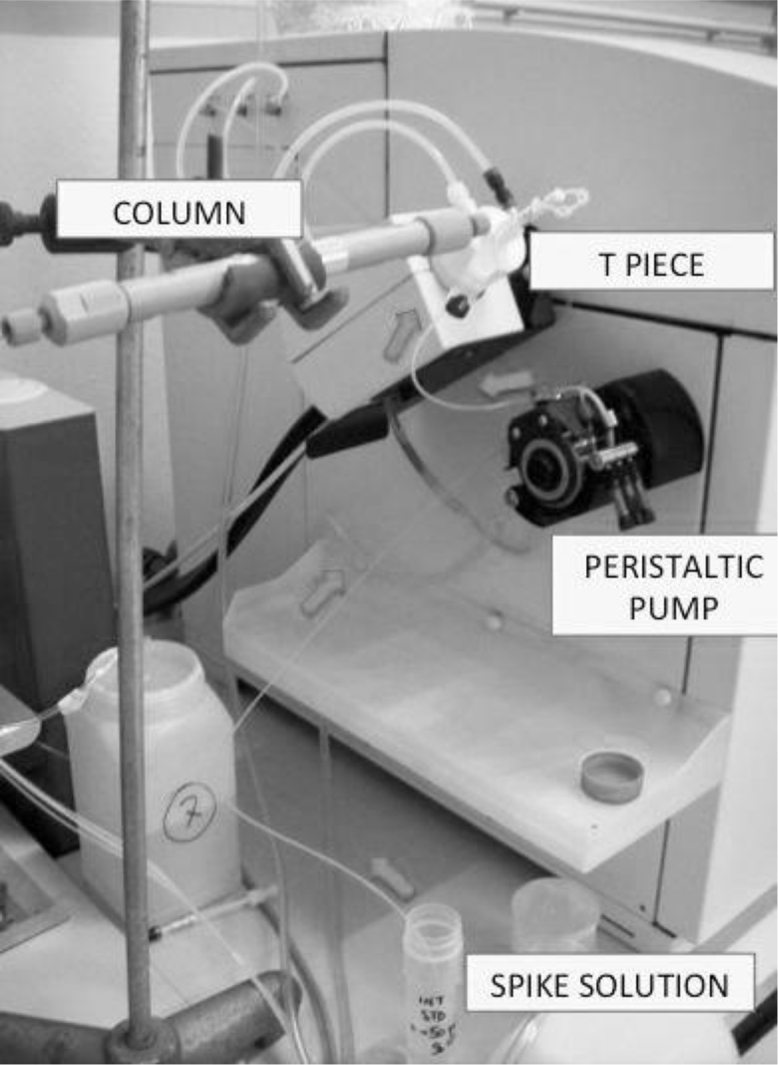
Representation of mix of spike solution and effluent from the column.

The diffusion coefficients of each inorganic species, measured in laboratory using DGT devices in aqueous solution with know concentration of Sb species at pH 5 and 0.01 M, were 7.60 ± 0.05 10^−6^ cm^2^ s^−1^ for Sb(III) and 5.23 ± 0.02 7.60 ± 0.05 10^−6^ cm^2^ s^−1^ for Sb(V) (see SUPPLEMENTARY INFORMATION, Table 2S and Fig. 2S). These values reflect the ionic characteristics of antimony species [2] and they were applied in DGT equation (Eq. (2)) for Sb species determination aqueous solutions.

The effects of pH and ionic strength were observed. At higher pH, measurements of Sb species in Fe oxide gel agreed with the solution concentrations (see SUPPLEMENTARY INFORMATION, Fig. 4S).

Strong negative effects on Sb species accumulation, especially for Sb(III), by resin gel of DGT were observed when electrolyte concentration was particularly low or absent (see SUPPLEMENTARY INFORMATION, Fig. 3S).

For one day deployment the Method Detection Limit (MDL) for a typical DGT device (0.78 mm thick diffusive gel, 0.13 mm filter) were 0.2 ng mL^−1^ for Sb(V) and 0.4 ng mL^−1^ for Sb(III).

## DGT devices application

### Materials

- Stock solutions of antimony species (1000 mg l^−1^ for Sb) prepared by antimony (III) potassium tartrate hemihydrate (C_4_H_4_KO_7_Sb · ½H_2_O), potassium hexahydroxoantimonate (KSb(OH)_6_) (Carlo Erba Reagents).
- Ultra-pure water prepared by a Milli-Q system (18 MΩ-cm resistance, Millipore® system, Millipore, Bedford, MA).
- Boxes for sampling waters with holders for DGTs devices.
- Iron (Fe) oxide DGTs (0.60 mm Fe-oxide gel, 0.78 mm open pore diffusive gel) (DGT Research Ltd., Lancaster, UK).
- Stirrer.
- Thermostatic chamber.
- Timer.

Solutions were prepared before DGT applications in the following way: 2.5 l of aqueous solution were mixed with know solutions of each species of inorganic antimony and they were well-stirred at constant temperatures in a cleaned box (Fig. 1).

The temperature of water should be measured at the beginning and the end of the application of DGTs.

At least three DGTs should be included in the box and they should be left about 24 h (the time should be exactly measured with timer). Time of contact and temperature were fundamental values to calculate the concentration of Sb(III) and Sb(V) in water after antimony extraction from resin gel of DGT through the equation based on Fick's first law of diffusion (Eq. (2))

After 24 h on the stirrer, DGT units were taken out of the solution and the surfaces were rinsed with ultra-pure water. After that, the resin gel could be retrieved and the Fe-oxide gel placed in a clean sample tube.

## Antimony species extraction

### Materials

- Plastic flasks (digiTUBES 50 ml).
- 10 mL of 50 mM of Ethylenediaminetetraacetic acid disodium salt dehydrate (C_10_H_14_N_2_Na_2_O_8_ · 2H_2_O) (Sigma Aldrich Co).
- Heating block system (DIGIPREP, Scp Science, Quebec, Canada).
- Ultra-pure water prepared by a Milli-Q system (18 MΩ-cm resistance, Millipore® system, Millipore, Bedford, MA).
- 0.45 μm filter (digiFILTER).

The Fe-oxide resin gels were placed in a plastic flask with 10 ml of 50 mM of Ethylenediaminetetraacetic acid disodium salt dihydrate. After that, they were mineralized at 95 °C for 90 min in a heating block system. The digested DGT gel solutions were filtered by using 0.45 μm filter (digiFILTER) after appropriate dilution with ultra-pure Water.

## HPLC-ID-ICP MS analysis

### Instrumentation and material

- ICP-MS (Agilent 7900, Agilent Technologies, USA) with Octopole Reaction System (ORS system) (Table 1).
- HPLC (Agilent 1100, Agilent Technologies, USA) (Table 1).
- HAMILTON PRP-X100 Anion exchange column (250 mm × 4.6 mm, 5 μm particle size).
- The standard solutions of antimony species were used by diluting the corresponding stock solutions.
- ^123^Sb-enriched standard solution (ISC Science, Oviedo, Spain) (Table 2).

**Fig. 3:**
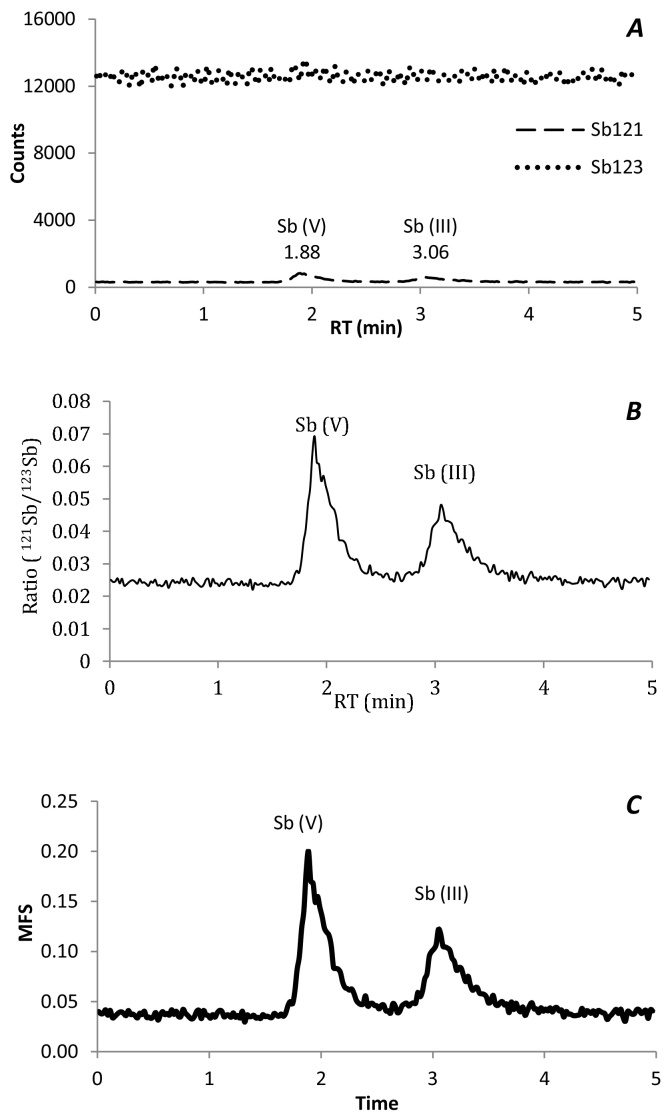
Chromatograms procedure to obtain mass flow with isotope dilution equation: 10 ng ml^−1^ of Sb(III) and Sb(V) in water (pH = 5 and 0.01 M NaNO_3_). A = isotope intensities chromatogram; B = chromatogram of ratio between isotope intensities; C = mass flow chromatogram.

Calibration points were prepared with the same percentage of EDTA in samples. In order to obtain the concentration of different species, we performed isotopic dilution analysis of calibration points under species-unspecific spiking. Continuous addition of spike solution of ^123^Sb, like enriched isotope, was carried out by peristaltic pump in such a way that was completely and continuously mixed – through a T piece – with eluent from the column with the separated species (Fig. 2). The number of mols of the Sb species was providing by integration of each chromatographic peaks in the molar flow chromatogram. The process to obtain the mass flow chromatogram (Fig. 3C) required the use of spreadsheet software. The row chromatograms should be available in table form with three columns: time, intensity of ^121^Sb and intensity of ^123^Sb, the graphic representation of each isotopes is shown in Fig. 3A. Then the isotope ratio, *R*_*m*_, was calculated like a time-depending function (Fig. 3B). The molar concentrations were transformed to mass concentrations using atomic weights with application of Eq. (1) [3].

(1) $$MF_{S}=c_{Sp}d_{Sp}f_{Sp}\frac{AW_{s}}{AW_{sp}}\frac{A_{sp}^{b}}{A_{s}^{a}}\left(\frac{R_{m}-R_{sp}}{1-R_{m}R_{s}}\right)$$

*MF*_*S*_ = mass flow of the sample eluting from the column

*c*_*Sp*_ = concentrations of the element in the spike (ex. 49.4546 ng/g)

*a* is the most abundant isotope in the sample

*b* is the most abundant isotope in the spike

*d*_*Sp*_ = density of spike solution (ex. 1 g ml^−1^)

*f*_*Sp*_ = flow rate of spike solution (ex. 0.04 ml min^−1^)

*AW*_*S*_ and *AW*_*Sp*_ = atomic weight of the element in the sample and in the spike

$A_{s}^{a}$ = Isotope abundances for isotopes *a* (121) in the sample (ex. 57.21)

$A_{sp}^{a}$ = Isotope abundances for isotopes *b* (123) in the spike (ex. 98.66)

*R*_*m*_ = the isotope ratio (a/b) (121/123) in the mixture

*R*_*Sp*_ = is the isotope ratio (a/b) (121/123, 1.343%/98.6575%) in the spike

*R*_*S*_ = the isotope ratio (b/a) (123/121, 42.79%/57.21%) in the sample

After that, the concentration of compounds at the corresponding retention time in samples was calculated by dividing the mass concentrations by sample volume injected. For a proper validation of the results, total analysis on samples should be realized to compare the values with calculated area from the whole chromatogram.

After that the concentration of the chemical forms of antimony absorbed by the resin of DGTs was quantified through the mass flow chromatogram, the time–averaged concentration (c) of each species in a solution was then calculated using DGT equation (Eq. (2)) [4].

(2) $$C_{DGT}=\frac{\left(M\times \Delta g\right)}{\left(t\times A\times D\right)}$$

Δ*g* = the thickness of the diffusive gel (cm),

*t* = the deployment time (s)

*A* = the surface area of the diffusive gel exposed to the bulk solution (cm^2^)

*D* = the diffusion coefficient of analyte in the diffusive gel (cm^2^ s^−1^),

*C*_*DGT*_ was compared with the immersion solution concentration analyzed in the samples taken during the experiment.

## Additional information

Antimony (Sb) is considered to be a nonessential element in plants, animals or humans [2], [5]. The US Environmental Protection Agency of the United States and the European Union evaluated Sb and its compounds as priority pollutants [6], [7].

In antimony compounds, the most common oxidation states are 5, 3, and -3. It exists mainly as Sb(III) and Sb(V) in environmental, biological, and geochemical samples.

Generally trivalent Sb compounds exert a 10 times higher acute toxicity than pentavalent Sb species. Its concentrations are much higher in natural geothermal systems, where they can range from 500 mg L^−1^ up to 10 wt.% [8], [9], [10], [11].

In this work we developed an analytical chemical procedure based on the above capability of using HPLC-ID-ICP-MS to separate and determine Sb(III) and Sb(V) in aqueous samples and Fe-oxide resins from DGT devices application.

The DGT technique is based on a simple device, which accumulates solutes on a binding agent after passage through a hydrogel, which acts as a well-defined diffusion layer [4]. Concentration of total dissolved metals in solution is calculated using Fick's first law of diffusion and the measured mass of solutes, accumulated on the binding agent after a known deployment time (Eq. (2)). In this way, we should measure those metal species that are available to organisms. These applications are a promising preservation procedure and they have the benefit of eliminating the risk of speciation changes due to transportation and storage of water samples prior to preparation and analysis. Moreover, this method of passive sampling can be used both in surface water that groundwater because the analyte uptake efficiency by resin is independent from pH and ionic strength – only low concentration of electrolyte can influence the behaviour of Sb species, especially Sb(III) – demonstrating that the charge of analyte or building agent does not significantly affect the uptake efficiency across environmental variables studied. Further studies on the interaction between Sb species and organic matter will be needed to observe the creation of complexes with function groups of humic compounds and their influence on the absorption efficiency of the antimony.

Isotope dilution analysis (IDA) is a well-known analytical technique based on the measurement of isotope ratios in samples, where its isotopic composition has been altered by the addition of a known amount of an isotopically enriched element (spike). HPLC-ICP-MS system allows to realize IDA thanks to its capability to perform isotope ratio measurements and consequently isotope dilution mass spectrometry due to the mass-specific detection system [12].

In our case, we applied on-line species-unspecific spiking mode. The addition of the isotope tracer or spike is carried out after the complete separation of the naturally occurring species in the sample has taken place (post-column spiking). This mode is especially useful either when the structure and composition of the species is not exactly known or when the corresponding isotopically labelled compounds are not commercially available or cannot be synthesis [3].

IDA reduces errors derived from instrumental instabilities and matrix effects providing accurate and precise determinations of elements. It is important that measured isotope ratios are corrected for mass bias and spectral interferences [13]. Instead use of ORC (octapole reaction system), in ICP MS analysis, overcome polyatomic interferences thanks to collisions produced by gas (He) between plasma and the quadrupole mass analyser [13].

## Figures and Tables

**Table 1:** Instrumental operating conditions of HPLC–ICP-MS.

Chromatographic conditions	
Column:	HAMILTON PRP-X100 Anion exchange column (250 × 4.6 mm, 5 μm particle size).
Eluent:	10 mM Na_2_EDTA; 1 mM of KHP
Flow rate:	1 ml min^−1^
Spike	Sb standard solution enriched in ^123^Sb (ISC Science, Oviedo Spain)
Flow rate spike	0.04 ml min^−1^
Injection volume:	10 μL
Column temperature	room temperature
Acquisition time	300 s

ICP-MS conditions	
Rf power:	1550 W
RF Matching:	1.8 V
Carrier gas flow rate:	Ar 1.05 l min^−1^
Dilution Mode:	ON
Dilution Gas:	Ar 0.2 l min^−1^
Sampling depth:	9 mm
S/C Temp:	2 °C
Reaction mode:	ON (He: 3 ml min^−1^)
Measured *m*/*z*:	Sb 121; Sb 123

**Table 2:** Characteristics of ^123^Sb-enriched standard solution (ISC Science, Oviedo, Spain).

^123^Sb-enriched standard solution product details:	
Chemical species:	Antimony nitrate
Isotope:	^123^Sb (98.66%)
Form:	2 ml in HNO_3_ (2%)
Isotope abundance (%) of ^121^Sb	1.343%
Isotope abundance (%) of ^123^Sb	98.657%
Concentration:	8.831 ± 0.196 (μg g^−1^ as Sb)
