# Supplementary material for: Determination of Sb(III) and Sb(V) by HPLC—Online isotopic dilution—ICP MS
Source: MethodsX. 2015 Dec 12;3:102–9. doi: 10.1016/j.mex.2015.12.001 (PMC4929249; doi:10.1016/j.mex.2015.12.001)
Supplement: Supplementary file 1 [file mmc1.pdf]

# Determination of Sb(III) and Sb(V) by HPLC – Online Isotopic Dilution – ICP MS.

Maria Chiara FONTANELLA[(1) (*)], Gian Maria BEONE[(1)], Ilenia CATTANI[(1)]

(1) Università Cattolica del Sacro Cuore, Istituto di Chimica Agraria e Ambientale, Via E. Parmense 84, 29100 Piacenza, Italy

(*) corresponding author. e-mail: mariachiara.fontanella@unicatt.it, phone number: +390523599212, cell number: +393487823028

**KINETICS OF BINDING AND ELUTION EFFICIENCY**

Triplicate iron (Fe)-oxide gel discs (3.14 $cm^2$ diameter, 0.60 mm of thickness) were placed in 10 mL of 50 µg $L^{-1}$ of antimony (Sb) inorganic species with a matrix of 0.01 M of $NaNO_3$ and they were loaded for various times from 0.5 min to 24h (Fig. 1S). Elution efficiency of these species was obtained by eluting loaded Fe-oxide gel discs in 50 mM of Ethylenediaminetetraacetic acid disodium salt dihydrate at 95°C for 90 min in heating block system (DIGIPREP, Scp Science, Quebec, Canada) in plastic flasks (digiTUBES 50 mL) (Table 1S). The elution and the immersion solutions were analysed by HPLC-ICP-MS with appropriate dilution.

Tab. 1S. Elution efficiencies of Sb(III) and Sb(V) determined for Fe-oxide gels using 10 mM $EDTA_3$. The error limits are standard deviations calculated for 10 replicates.

| | Sb(III) | Sb(V) |
|---|---|---|
| 10 mM EDTA | 1.03 ± 0.11 | 1.03 ± 0.05 |

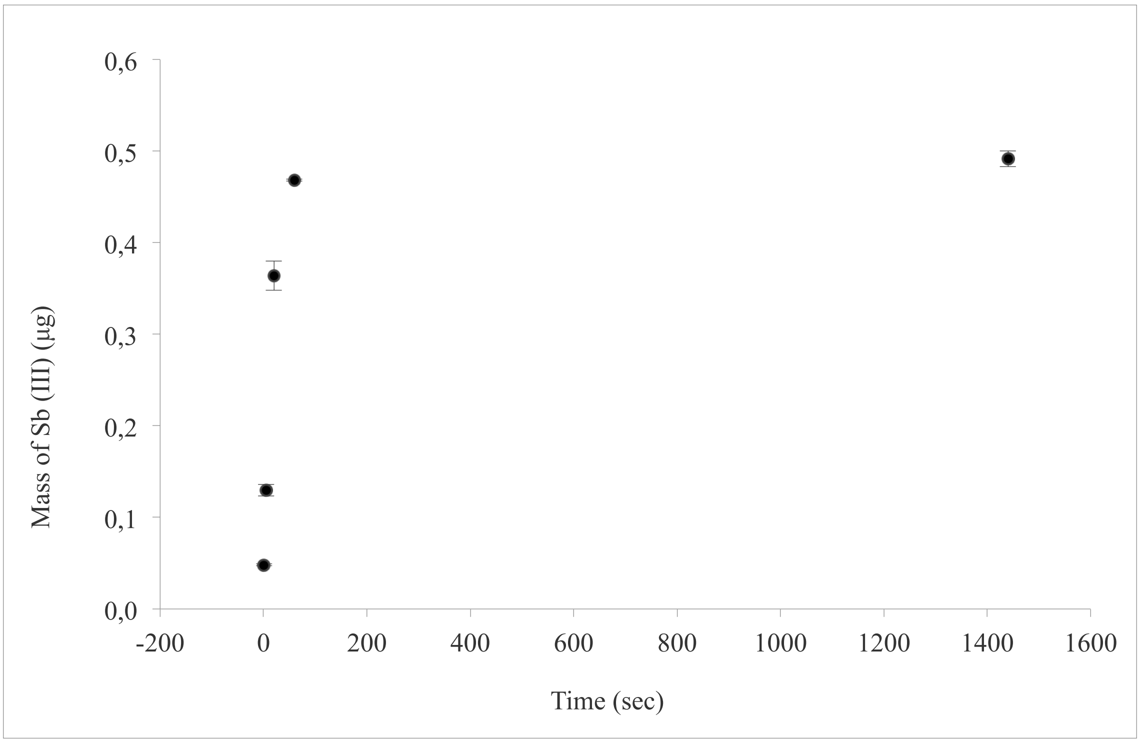

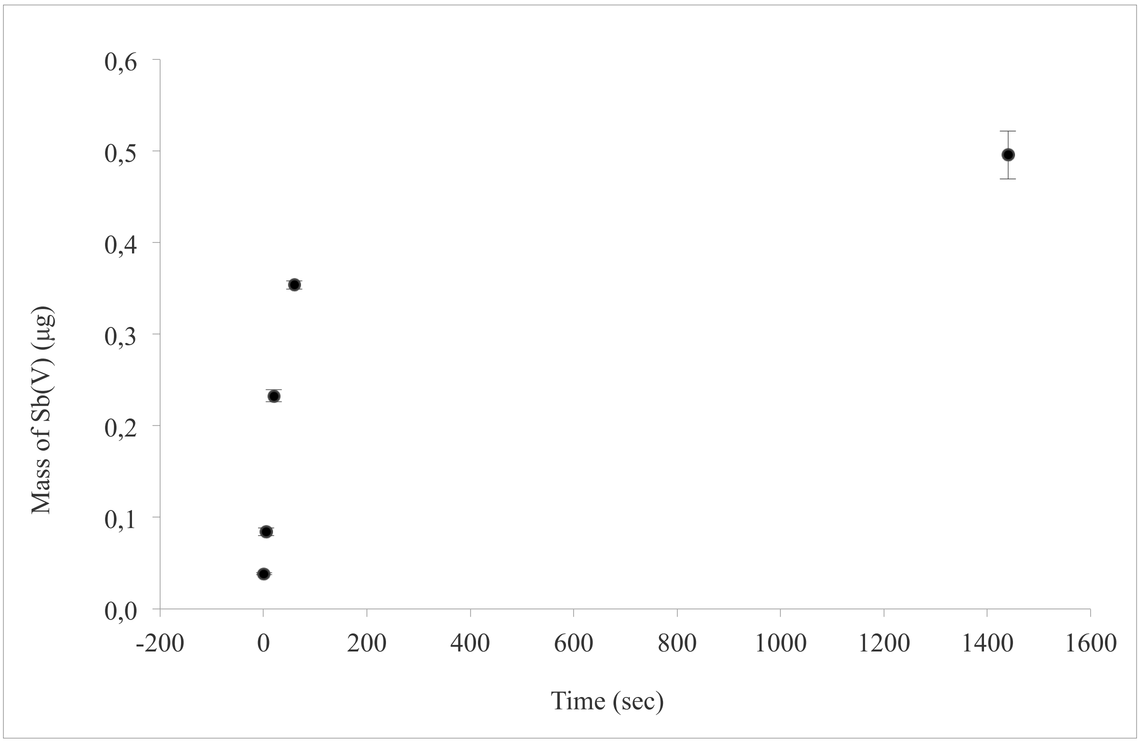


Fig. 1S. Mass of Sb(III) and Sb(V) accumulated by Fe-oxide gels placed in solutions containing 50 ugr $l^{-1}$ Sb for different times. Error bars are calculated from the standard deviation of replicates (n=3).

## MEASUREMENT OF Sb DIFFUSION COEFFICIENTS USING DGT DEVICES

Diffusion coefficients of Sb(V) and Sb(III) were measured using DGT devices. Ten DGT devices were deployed in the Sb solution for times ranging from 3 to 24 h. At each retrieval time, 2 DGT devices were removed providing duplicate samples (Fig. 2S). The diffusion coefficients were calculated using Eq. 1S.

$$D = \frac{(slope \times \Delta g)}{(C \times A)} \qquad \text{(Eq. 1S)}$$

Where *D* is the diffusion coefficient, *slope* is the slope of a linear plot of measured mass of Sb diffused through the diffusive gel (and membrane filter) vs. time, *A* is the exposed area of diffusive gel/membrane filter, *Δg* is the combined thickness of the diffusive gel and membrane filter (i.e. 0.091 cm), and *C* is the concentration of Sb initially present in the source compartment of the

diffusion cell. At the start and end of experiment and at each retrieval time, the concentration of Sb in the source compartment was measured to confirm that the Sb species concentration did not change significantly over the experimental time.

This procedure was also made to measure and control changes in pH values and temperature. Corrections for measurements made at different temperatures were made using the equation applied by Zhang and Davison [1]. This equation was used to obtain D values at 24 ± 0.5°C needed for calculations of $C_{DGT}$ when DGT was deployed in different synthetic solutions (Table 2S).

Tab. 2S. Diffusion coefficients $10^{-6}$ $cm^2$ $s^{-1}$ measured using DGT devices at pH 5. Valid at 24 ± 0.5 °C. Uncertainty is the S.D. of the mean from *n* replicate determinations.

| **Sb species** | DGT devices | Literature |
|---|---|---|
| Sb(III) | 7.60 ± 0.05 | |
| | | 5.4 [2] |
| Sb(V) | 5.23 ± 0.02 | 5.38 [3]. |
| | | 6.86 [4] |

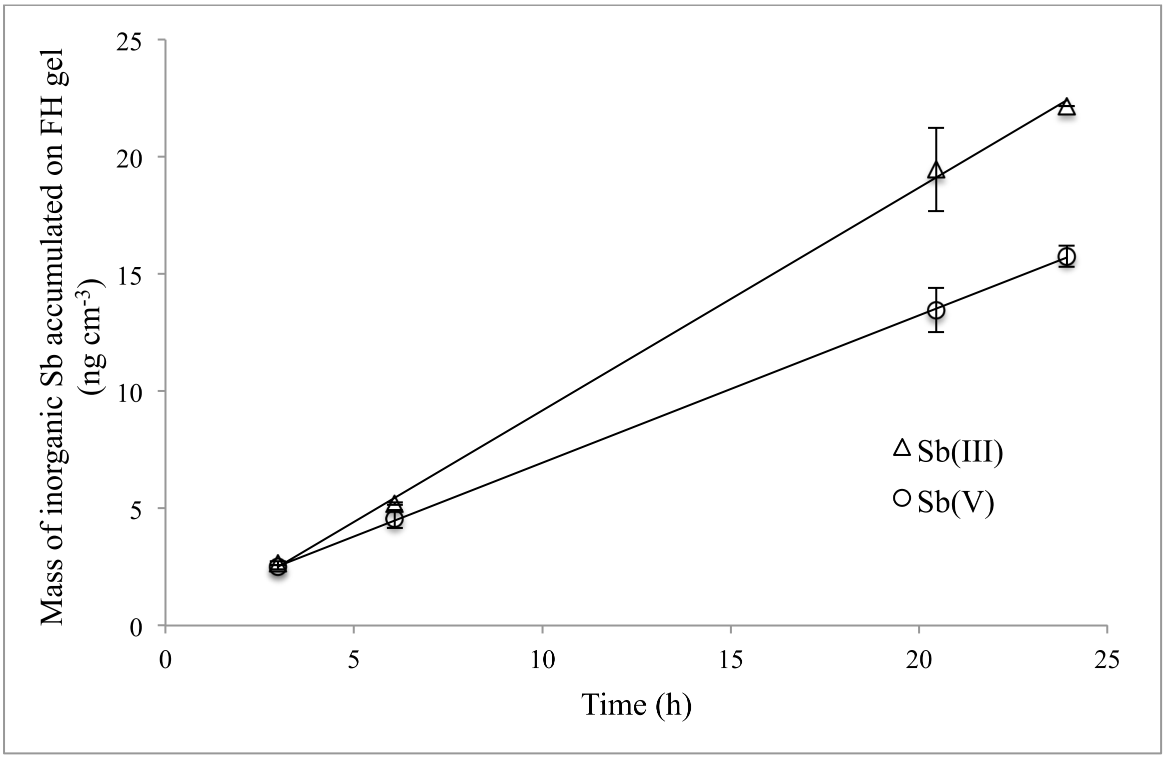


Fig. 2S. Plots of measured mass of inorganic species of antimony.

**IONIC STRENGTH AND pH INFLUENCE**

Triplicate FH DGTs (0.60 mm Fe-oxide gel, 0.78 mm open pore diffusive gel), were deployed for 24h in various well-stirred solutions at known constant temperatures to test the effects of ionic strength and pH

To study the effect of ionic strength, a range of $NaNO_3$ concentration, from 0 to 1 mM, was prepared in 2.5 l of solution (pH 5.13 ± 0.11) containing each species at 50 µg $l^{-1}$ (Fig. 3S).

For pH influence, 2.5 l containing 0.01 M were prepared at different pHs adjusted with diluted NaOH prior to spiking with Sb species stock solutions to 50 µg $l^{-1}$ (Fig. 4S).

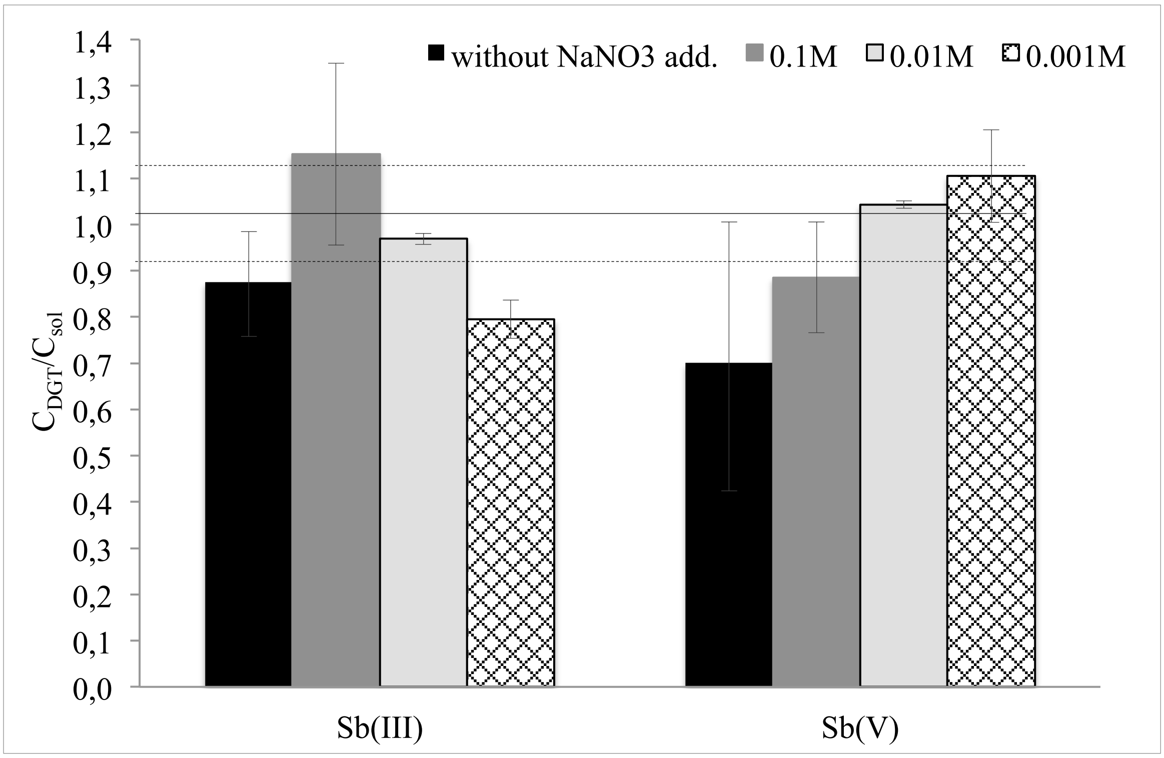


Fig. 3S. Effect of concentration of supporting electrolyte, $NaNO_3$, on the ratio of concentrations measured by DGT containing Fe-oxide gels, $C_{DGT}$, to deployment solution concentrations of Sb(III) and Sb(V), $C_{sol}$. Error bars represent the standard deviation of three replicates. The solid horizontal line and dotted horizontal lines represent target values of 1 ± 0.1.

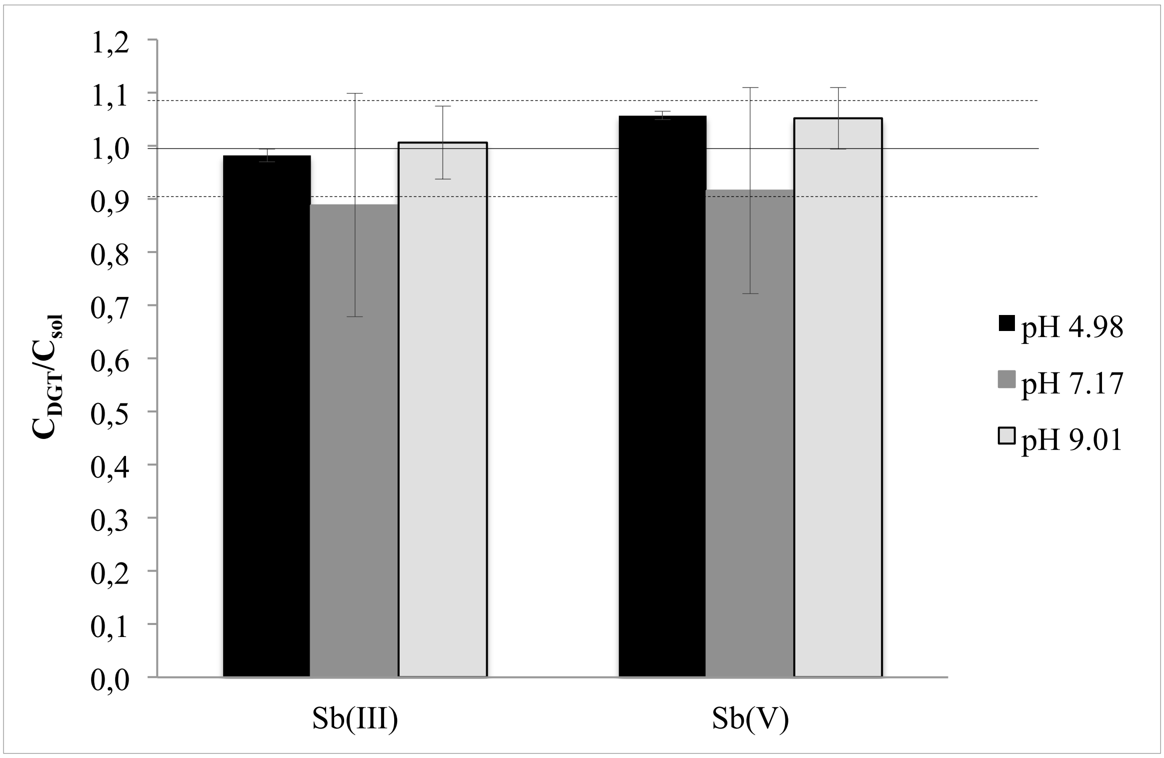


Fig. 4S. Effect of pH on the ratio of concentrations of Sb(III) and Sb(V), measured by DGT, $C_{DGT}$, to deployment solution concentrations, $C_{sol}$. These measurements were performed in the presence of 0.01 M $NaNO_3$. Error bars represent the standard deviation of three replicates. The solid horizontal line and dotted horizontal lines represent target values of 1 ± 0.1.

## REFERENCES

[1] H. Zhang and W. Davison. 1995. Performance characteristics of diffusion gradients in thin films for the in situ measurement of trace metals in aqueous solution. Anal. Chem. 67, 3391-3400. doi: 10.1021/ac00115a005

[2] J. Luo, H. Zhang, J. Santner and W. Davison. 2010. Performance characteristics of diffusive gradients in thin films equipped with a binding gel layer containing precipitated ferrihydrite for measuring arsenic(V), selenium(VI), vanadium(V), and antimony(V). Anal. Chem. 82, 8903-8909. doi: 10.1021/ac101676w

[3] H. Osterlund, S. Chlot, M. Faarinen, A. Widerlund, I. Rodushkin, J. Ingri, D.C. Baxter. 2010. Simultaneous measurements of As, Mo, Sb, V and W using a ferrihydrite diffusive gradients in thin films (DGT) device. Anal. Chim. Acta 682, 59-65. doi:10.1016/j.aca.2010.09.049

[4] JG. Panther, RR. Stewart, PR. Teasdale, WW. Bennett, DT. Welsh, H. Zhao. 2013. Titanium dioxide-based DGT for measuring dissolved As(V), V(V), Sb(V), Mo(VI) and W(VI) in water. Talanta 105, 80–86. doi:10.1016/j.talanta.2012.11.070
